# High Levels of Soluble C5b-9 Complex in Dialysis Fluid May Predict Poor Prognosis in Peritonitis in Peritoneal Dialysis Patients

**DOI:** 10.1371/journal.pone.0169111

**Published:** 2017-01-03

**Authors:** Masashi Mizuno, Yasuhiro Suzuki, Keiko Higashide, Yumi Sei, Daiki Iguchi, Fumiko Sakata, Masanobu Horie, Shoichi Maruyama, Seiichi Matsuo, B. Paul Morgan, Yasuhiko Ito

**Affiliations:** 1 Renal Replacement Therapy, Nagoya University Graduate School of Medicine, Nagoya, Japan; 2 Division of Nephrology, Nagoya University Graduate School of Medicine, Nagoya, Japan; 3 Daiyukai-daiichi Hospital, Ichinomiya, Japan; 4 Complement Biology Group, Division of Infection and Immunity, School of Medicine, Cardiff University, Cardiff, United Kingdom; University of Leicester, UNITED KINGDOM

## Abstract

**Background:**

We searched for indicators to predict the prognosis of infectious peritonitis by measuring levels of complement proteins and activation products in peritoneal dialysis (PD) fluid (PDF) of patients at early stages of peritonitis. We retrospectively analyzed the relationship between the levels of sC5b-9, C3 and C4 in PDF and the subsequent clinical prognosis.

**Methods:**

We measured levels of sC5b-9, C3 and C4 in PDF on days 1, 2 and 5 post-onset of peritonitis in 104 episodes of infectious peritonitis in PD patients from 2008 and retrospectively compared levels with clinical outcomes. Further analysis for the presence of causative microorganisms or to demonstrate bacterial culture negative peritonitis was performed and correlated with change of levels of sC5b-9 in PDF.

**Results:**

When PD patients with peritonitis were divided into groups that either failed to recover from peritonitis and were finally withdrawn from PD (group 1; n = 25) or recovered (group 2; n = 79), levels of sC5b-9, C3 and C4 in PDF were significantly higher in group 1 patients compared to those in group 2 on day5. Analysis of microorganisms showed significantly higher sC5b-9 levels in PDF of peritonitis cases caused by culture negative peritonitis in group 1 compared with group 2 when we analyzed for individual microorganisms. Of note, on day5, the sC5b-9 levels in PDF were similarly high in peritonitis caused by fungi or other organisms.

**Conclusion:**

Our results suggested that levels of complement markers in PDF, especially sC5b-9, have potential as surrogate markers to predict prognosis of PD-related peritonitis.

## Introduction

Infectious peritonitis is an important complication that prevents long term peritoneal dialysis (PD) therapy in end-stage renal disease patients [1,2]. For peritonitis that does not respond to any antibiotic therapies, removal of the PD catheter is required. The International Society of Peritoneal Dialysis 2010 guideline recommends that the PD catheter is removed when peritonitis is not improved by 5 days after onset [3]. It is then usually necessary to transfer to hemodialysis (HD) in these patients; this requires surgical operations both to remove the PD catheter and to make the arteriovenous fistula for HD therapy. These events are very stressful for patients, leading clinicians to delay performance of these procedures if the patient does not have obvious symptoms of peritonitis. Markers of likely outcome would aid clinical decision-making and improve patient outcome.

The complement (C) activation system is an important defense system to protect the host from attack by invasive microorganisms. In contrast, excessive C activation can drive inflammation and damage host cells and tissues [4,5]. Therefore, it is important to maintain the balance between C activation and suppression. Tight regulation of the C system is required to maintain homeostasis and to prevent tissue injures in the host [4,5]. As we previously reported, membrane C regulators (CRegs) play key roles to maintain host homeostasis in the healthy peritoneum [6]. When inflammation was induced in the peritoneum in the rat model, inhibition of CRegs resulted in uncontrolled local C activation and enhanced peritoneal injuries [7,8]. Although C activation protects the host from invasion of microorganisms in peritoneum, unregulated activation of the C system destroys the peritoneum. In PD fluids (PDF) from patients, C proteins such as C3 and C4 were observed [9] and local production was shown from human peritoneal mesothelium under PD [10] suggesting capacity for control of inflammation/infection in peritoneum. We also reported that the CRegs CD46, CD55 and CD59 are broadly distributed in human peritoneum [11], suggesting that the balance of C activation and regulation might be important to maintain homeostasis in human peritoneal cavity. When PD-related peritonitis occurs, activation and/or dysregulation of the C system could be induced in the peritoneum. In this context, it is relevant to clarify detail of components and products of C activation in PD fluid during individual episodes of peritonitis in PD patients and the relationship between prognosis of peritonitis and generated C products. Therefore, we searched for indicators to predict the prognosis of infectious peritonitis by measuring levels of C components and activation products in PDF of PD patients at early stage of peritonitis, especially culture negative peritonitis.

## Materials and Methods

### PDF samples of PD patients

PDFs with overnight dwell were collected from 104 peritonitis cases on days 1, 2 and 5 post-onset from May 2008 to December 2013 in Nagoya University Hospital and Daiyukai Daiichi Hospital and Jan 2012 to December 2012 in Handa City Hospital. S1 Table shows number of PD patients, total number of peritonitis episodes and number of sample episodes in each institute during the periods. The present study was approved by the Ethics Committee for Human Research of the Faculty of Medicine at Nagaya University (Approval numbers 298, 309 and 2013–2075), subsequently by the ethical committees of Daiyukai-daiichi Hospital and Handa City Hospital. We used samples of patients who had provided their written agreements in the hospital to the use of their PDF and demographic/laboratory data in the present study. The ethical committees approves the above procedure. The basic patient profiles of each institute are shown in S1 Table. The collected PDF samples were centrifuged promptly and the supernatants were kept at -80°C until measurement of C proteins by enzyme-linked immunosorbent assays (ELISA).

We retrospectively collected data of total white blood cell count in PDF, serum levels of C-reactive protein, C3, C4 and CH50, and results of bacterial cultures of PDF from medical records. Peritonitis was diagnosed according to the 2010 guideline of the International Society for Peritoneal Dialysis (ISPD) [3]. We also collected laboratory data of levels of serum albumin (g/dL) and creatinine (mg/dL), blood urea nitrogen and hemoglobin (g/dL) within one month before each episode of peritonitis from the medical records.

To analyze prognosis, we divided the 104 peritonitis cases into two groups as follows; withdrawal from PD therapy caused by peritonitis (group1; n = 25); finally recovered from peritonitis (group2; n = 79).

### Methods of bacterial cultures from PD effluent

For bacterial cultures to identify the causative microorganisms, a combination of the following two methods, recommended in the 2010 ISPD guideline [3], were performed in Nagoya University Hospital and Handa City Hospital throughout the observational periods and in Daiyukai-daiichi Hospital from April 2011. In the first method, 50 mL PD effluent was centrifuged, the sediment was directly inoculated for culture on standard culture media. In the second method, 10mL of PD effluent was inoculated into standard blood-culture media under aerobic or anaerobic condition. Regarding patients presenting at the emergency room on bank holidays or at night, bacterial culture was limited to the second method only because of logistic issues. PD patients with peritonitis presenting in Daiyukai-daiichi Hospital were also only tested using the second method until March 2011.

### Measurements of levels of total protein, soluble C5b-9 (sC5b-9), C3 and C4 in PD fluids

To measure levels of sC5b-9, C3 and C4 in PDF, we used the MicroVue^TM^ sC5b-9 Plus EIA kit (Quidel Co., San Diego, CA), AssayMax Human Complement C3 ELISA kit and AssayMax Human Complement C4 ELISA kit (Assaypro LLC, St. Charles, MO), respectively. These measurements were performed according to the manufacturer’s instructions. Total protein amounts in PDF were also measured by BCA protein assay reagent (Thermo Fisher Scientific) to adjust levels of sC5b-9, C3 and C4 in PDF. Firstly, we simply measured sC5b-9 levels in PDF and compared the raw data with total protein amounts in PDF and white blood cell (WBC) counts in PDF. Secondly, each complement analyte level was adjusted by the total protein level of PDF as in the example below;

Adjusted sC5b-9 level in PDF (ng/mg) = (sC5b-9 level in PDF (ng/mL)) / (protein level in PDF (mg/mL)

Adjusted C3 or C4 level in PDF (μg/mg) = (C3 or C4 level in PDF (μg/mL)) / (protein level in PDF (mg/mL)

Percent change of sC5b-9 levels in PDF was also calculated as below;

sC5b-9 (D2-D1)/D1 (%) = {(adjusted sC5b-9 level in PDF on day 2)–(adjusted sC5b-9 level in PDF on day1)} / (adjusted sC5b-9 level in PDF on day1) × 100

sC5b-9 (D5-D1)/D1 (%) = {(adjusted sC5b-9 level in PDF on day 5)–(adjusted sC5b-9 level in PDF on day1)} / (adjusted sC5b-9 level in PDF on day1) × 100

sC5b-9 (D5-D2)/D2 (%) = {(adjusted sC5b-9 level in PDF on day 5)–(adjusted sC5b-9 level in PDF on day2)} / (adjusted sC5b-9 level in PDF on day2) × 100

### Relationship between and time course of adjusted sC5b-9 and WBC counts in PDF

We compared adjusted sC5b-9 levels in PDF and WBC counts in PDF between groups 1 and 2 on days 1, 2 and 5. We further performed multivariate logistic regression analysis to statistically evaluate the predictive ability of those biomarkers on day 5 for subsequent clinical outcomes.

### Statistical analysis

Data was summarized as median (interquartile range) for continuous variables and number (%) for categorical variables. Baseline data was compared between group 1 and group 2 with Mann-Whitney U test or Fisher’s exact test as appropriate.

Spearman’s rank correlation coefficients were calculated to examine correlation between adjusted sC5b-9 levels in PDF with counts of WBC in PDF.

Intergroup comparison was performed using Kruskal-Wallis test, followed by Mann-Whitney U test for post-hoc pairwise comparisons. Intragroup comparison was performed using Friedman test, followed by Wilcoxon signed-rank test for pairwise comparison with day 1. To address the multiplicity of comparison, Bonferroni method was applied to calculate corrected P-values.

Using multivariate logistic regression models, we examined whether the prediction capability of group 1 is improved by the addition of sC5b-9, C3, or C4 at day 5 to the model consisting of a conventional predictive factor, WBC (Model 1). Baseline imbalance in patient’s characteristics was adjusted in the models. We evaluated the improvement of the prediction capability by calculating concordance index (c-index), net reclassification improvement (NRI), and integrated discrimination improvement (IDI). To predict withdrawal from PD because of peritonitis, threshold value of adjusted sC5b-9 level in PDF was calculated by receiver operating characteristics analysis to estimate optimal cut-off value.

A two-sided *P*-value of <0.05 was considered significant. Statistical analyses were carried out using IBM SPSS Statistics 23.0 (International Business Machines Corp, Armonk, NY) and R version 3.2.2 (R Foundation for Statistical Computing, Vienna, Austria).

## Results

### Comparison of background and basic data

Comparison of the background patient characteristics between Groups 1 and 2, age, gender, and status of diabetes mellitus as a background disease, revealed no significant differences. Duration of PD therapy prior to the episode of peritonitis was slightly but significantly longer in group 1 than in group 2.

Serum creatinine levels and blood urea nitrogen level measured within one month before the episode of peritonitis were not significantly different between groups 1 and 2. Serum albumin levels in group 1 tended to be lower compared with group 2. No significant differences in serum levels of C3, C4, CH50, and WBC counts in PDF, were observed between groups 1 and 2. These background data are summarized in Table 1.

**Table 1:** Comparison of Background of patients.

	Group 1	Group 2	*P*-value^a^
Number of peritonitis episodes	25	79	
Age (years)	67 (58, 75)	67 (60, 73)	0.684
Male/Female	22 (88.0)/3 (12.0)	59 (74.7)/20 (25.3)	0.268
DM/non DM	11 (44.0)/14 (56.0)	39 (49.4)/40 (50.6)	0.655
PD history (month)	46 (21, 69)	24 (10, 45)	0.013*
Serum albumin level (g/dL)	2.9 (2.5, 3.4)	3.3 (2.9, 3.6)	0.067
Blood urea nitrogen level (mg/dL)	52.1 (40.0, 62.7)	48.0 (39.0, 59.0)	0.396
Serum creatinine level (mg/dL)	8.40 (7.00, 10.41)	8.08 (7.05, 9.86)	0.670
Serum C3 level (mg/dL)	86 (84, 102)	94 (84, 111)	0.225
Serum C4 level (mg/dL)	27 (24, 33)	30 (27, 34)	0.272
Serum CH50 (U)	43.5 (37.6, 52.0)	42.7 (39.6, 50.8)	0.650

Notes: Data are shown as median (interquartile range) or number (%). Raw data is shown in S2 Table.

^a^Mann-Whitney U test for continuous data, Fisher’s exact test for categorical data.

*P<0.05

In the 104 peritonitis episodes, causative microorganisms that could be evaluated are listed in Table 2; 33.7% of the cases were culture negative under bacterial examination. The majority of culture-positive infections were with gram positive microorganisms but there were multiple species observed. The distribution of microorganism species was similar to that recorded in our local PD registry [1].

**Table 2:** List of causative organisms in peritonitis.

n (%)	Microorganisms	Group 1	Group 2
Gram positives		6 (24.0)	37 (46.8)
	*Streptococcus sp*.	0 (0)	13 (16.5)
	*Staphylococcus aureus*	2 (8.0)	5 (6.3)
	*coagulase-negative staphylococcus (CNS)*	3 (12.0)	11 (13.9)
	*Enterococcus sp*.	1 (4.0)	6 (7.6)
	*Micrococcus sp*.	0 (0)	1 (1.3)
	Un-classified	0 (0)	1 (1.3)
Gram negatives		10 (40.0)	11 (13.9)
	*Escherichia coli*	2 (8.0)	2 (2.5)
	*Pseudomonas aeruginosa*	3 (12.0)	0 (0)
	*Acinetobacter sp*.	0 (0)	6 (7.6)
	*Chryseobacterium sp*.	1 (4.0)	1 (1.3)
	*• Serratia sp*. *or Others*	4 (16.0)	2 (2.5)
	*• (Pseudomonas sp*.,		
	*• Klebsiella sp*.,		
	*• Bacteroides sp*.,		
	*• Alcaligenese sp*.,		
	*• un-classified)*		
Fungus		5 (20.0)	0 (0)
	*Candida sp*.	4 (16.0)	0 (0)
	*Cryptococcus sp*.	1 (4.0)	0 (0)
Culture negatives		4 (16.0)	31 (39.2)
Total		25 (100)	79 (100)

### Levels of sC5b-9 and protein in PDF from PD patients with peritonitis

**Fig 1:**
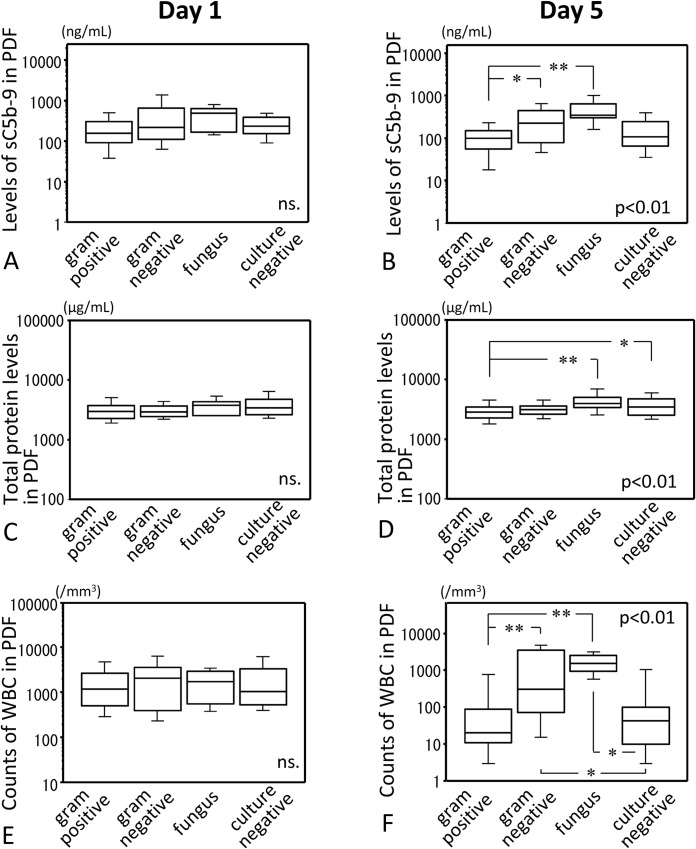
Measurements of sC5b-9, total protein and WBC in PDFs on peritonitis days 1 and 5. All cases were categorized into gram positives, gram negatives, fungal infection (fungus) and culture negative based on identified causative microorganisms. (A) and (B) show levels of sC5b-9 in peritoneal dialysates (PDFs); (C) and (D) show levels of total protein in PDF; (E) and (F) show white blood cells (WBC) counts in PDF. (A), (C) and (E), day 1 post-onset; (B), (D) and (F), day 5 post-onset. Statistical tests with Kruskal-Wallis test followed by Mann-Whitney U test and Bonferroni correction. *, p<0.05; **, p<0.01.

Levels of sC5b-9 in PDF were significantly correlated with those of protein and WBC counts in PDF on day 1, 2, and 5 (data not shown). When we compared sC5b-9 levels, protein levels and WBC counts in PDF among four categories based on causative microorganisms (gram positives, gram negatives, fungus, culture negative), significant differences among four categories were observed on day 5 but not on day 1 (Fig 1). Leakage of protein into PDF was significantly correlated with sC5b-9 levels in PDF. As previously reported [12,13], in non-inflamed peritoneum without peritonitis, leakage of proteins larger than α_2_-macroglobulin was minimal from blood to peritoneal cavity. The sC5b-9 complex has a molecular mass in excess of 10^6^ Da, more than that of α_2_-macroglobulin (750kDa), and is unlikely to significantly traverse the selective barrier of peritoneum, suggesting that measured sC5b-9 in PDF was mainly locally produced. However, components of sC5b-9 in PDF might be considered to leak from blood dependent on the level of total protein, because total protein level in PDF was well correlated with levels of sC5b-9 in PDF. This point may be supportive to follow corrected levels of sC5b-9 even if the uncorrected values might have the same predictive capability for outcome. Therefore, we adjusted sC5b-9 levels in PDF with protein levels in the PDF for the further analyses.

### Protein-adjusted levels of sC5b-9, C3 and C4 and counts of WBC in PDFs from PD patients with peritonitis

**Fig 2:**
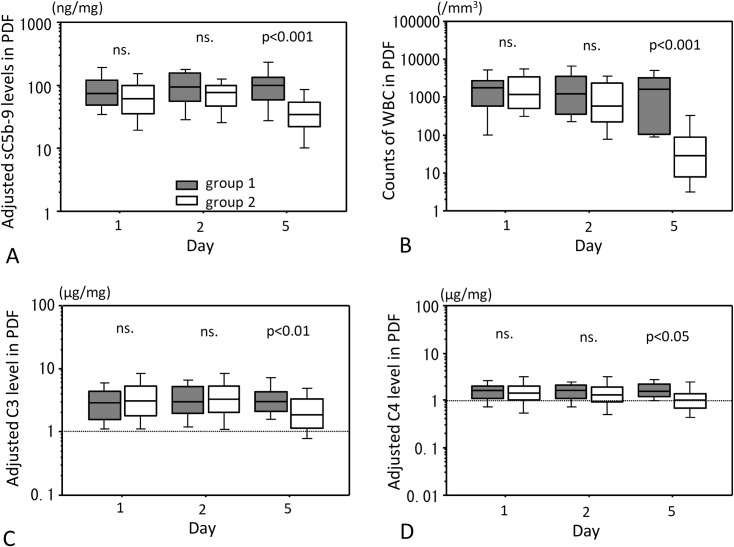
PDF levels of adjusted sC5b-9, C3 and C4 and WBC counts after onset of peritonitis. PDF levels of sC5b-9, C3 and C4 adjusted for total protein on Days 1, 2 and 5 after onset of peritonitis are shown in (A), (C) and (D), respectively. Counts of WBC in PDFs are shown in (B). Gray hatched bar is group 1 and open hatched bar is group 2. ns., not significant. Statistical tests with Mann-Whitney U test and Bonferroni correction.

Statistically significant differences of adjusted sC5b-9 levels, adjusted C3 levels, adjusted C4 levels and counts of WBC in PDF were observed between days 1, 2 and 5 in group 2 (p<0.001) but not in group 1. Compared with day 1, significant decreases in adjusted sC5b-9, C3 and C4 levels in PDF were observed on day 5 (p<0.001), and also in counts of WBC levels in PDF on days 2 and 5 (p<0.01 and p<0.001, respectively). There were no significant differences between group 1 and group 2 in levels of all complement markers in PDF on days 1 and 2 (Fig 2), whereas all of them were significantly lower in group 2 than in group 1 on day 5. WBC counts in PDF were also lower in group 2 than in group 1 on day 5. Because levels of adjusted sC5b-9, C3 and C4 in PDF were different between groups 1 and 2 on day 5, we further analyzed these markers focusing on day 5.

### Percent changes of adjusted sC5b-9, C3 and C4 in PDFs from PD patients with peritonitis

**Fig 3:**
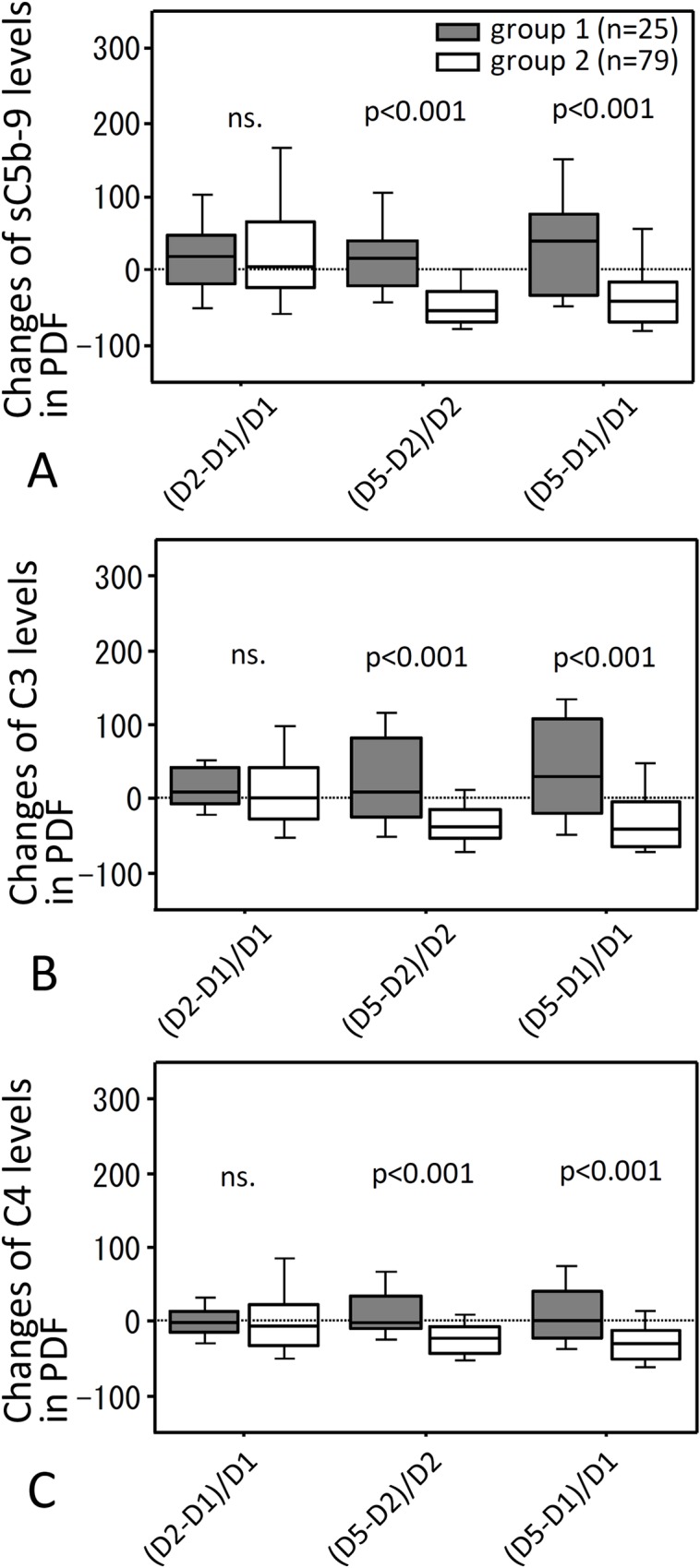
Percent change of the PDF levels of adjusted sC5b-9, C3 and C4 after onset of peritonitis. (A) shows percent change of adjusted levels in PDF of sC5b-9 (A), C3 (B) and C4 (C). (D2-D1)/D1, (D5-D2)/D2 or (D5-D1)/D1 shows changes from days 1 to 2, from days 2 and 5, or from day 1 to 5, respectively. Gray filled hatched bar is group 1 and open hatched bar is group 2. Statistical tests with Mann-Whitney U test and Bonferroni correction. ns., not significant.

Percentage changes of adjusted sC5b-9, C3 and C4 levels in PDF were not significantly different between groups 1 and 2 from day 1 to day 2, whereas they were significantly larger in group 1 from day 2 to day 5 and from day 1 to day 5 (Fig 3A, 3B and 3C).

### Levels of adjusted sC5b-9, C3 and C4 in PDF on day 5 for four categories of microorganisms causing peritonitis

**Fig 4:**
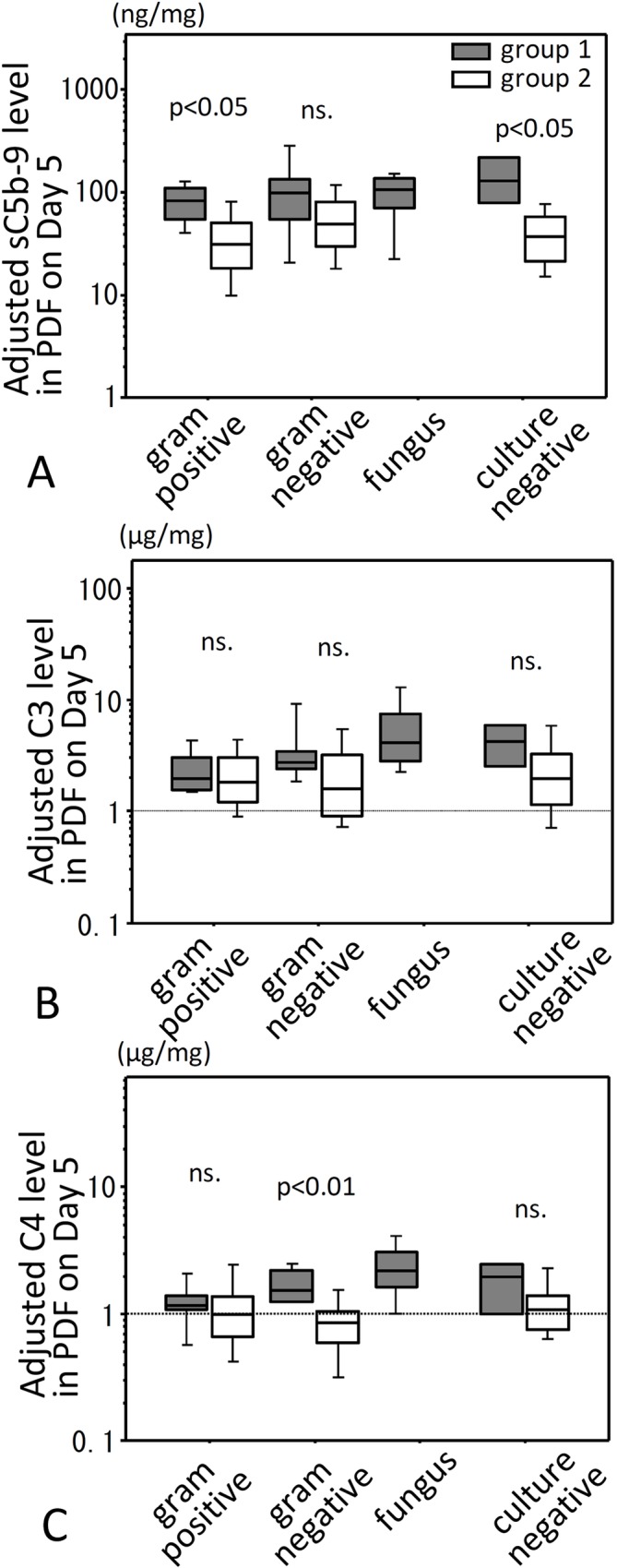
PDF levels of adjusted sC5b-9, C3 and C4 on Day 5 for four categories of microorganisms causing peritonitis. Levels of adjusted sC5b-9, C3 and C4 in PDF were compared for four categories of microorganisms causing peritonitis on Day 5 after onset of peritonitis. (A) shows levels of adjusted sC5b-9 in PDF, (B) shows levels of adjusted C3 in PDF, and (C) shows levels of adjusted C4 in PDF. Filled bar is group 1 and open bar is group 2. Statistical tests with Mann-Whitney U test and Bonfferoni correction. ns., not significant.

When bacteriological results were categorized into gram positive, gram negative, fungus or culture negative, adjusted levels of sC5b-9 in gram positive and culture negative PDF were significantly higher in group 1 than in group 2 (Fig 4A). No significant difference was observed in adjusted C3 levels between groups 1 and 2 (Fig 4B), while adjusted levels of C4 in gram negative infected PDF were significantly higher in group 1 compared to group 2 (Fig 4C). All cases with fungal peritonitis were withdrawn from PD according to recommendation of the ISPD guideline immediately after fungal infection was identified, therefore we could not statistically compare fungal peritonitis between groups 1 and 2. However, adjusted levels of sC5b-9, C3 and C4 were similarly high in fungal infected PDF in group 1as the other three categories in group 1.

### Levels of adjusted sC5b-9, C3 and C4 in PDF on Day 5 stratified by individual species of microorganisms causing peritonitis

**Fig 5:**
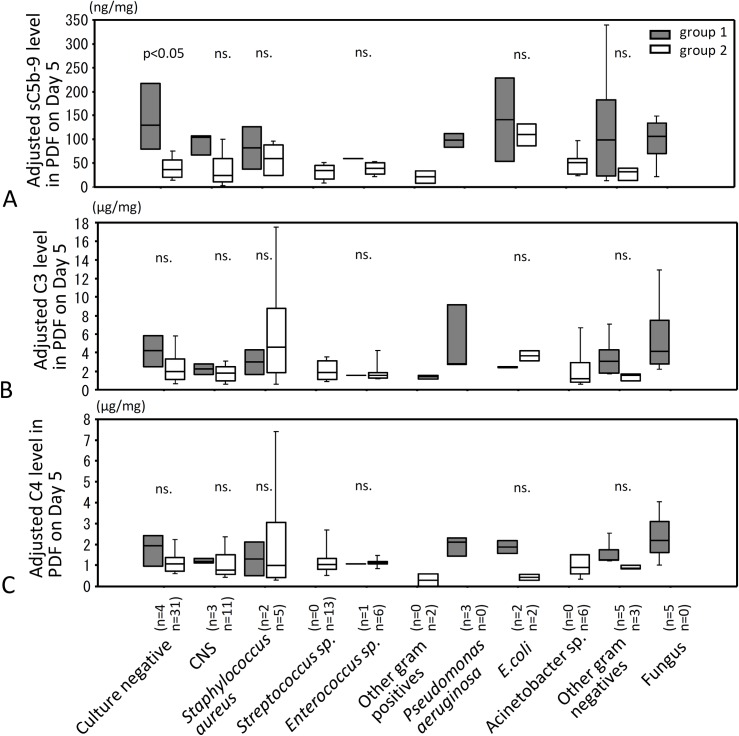
Levels of sC5b-9, C3 and C4 in PDF and relationship to species of microorganism. Levels of sC5b-9, C3 and C4 in PDF from patients with peritonitis on day 5 after onset of peritonitis and relationship to species of microorganism were investigated. *Staphylococcus aureus* includes both methicillin sensitive and methicillin resistant organisms. Gray filled hatched bar is group 1 and open hatched bar is group 2. Statistical tests with Man-Whitney U test and Bonferroni correction. Culture negative: no growth in bacterial culture examination, CNS: coagulase negative *Staphylococcus spp*., ns: not significance.

Culture positive peritonitis cases were categorized into *coagulase-negative staphylococci (*CNS), *Staphylococcus aureus*, *Streptococcus*, *Enterococcus*, other gram positives, *Pseudomonas aeruginosa*, *E*.*coli*, *Acinetobacter*, other gram negatives, or fungus and analyzed in the same way (Fig 5A); on day 5 adjusted sC5b-9 levels in culture negative PDF were significantly higher in withdrawal PD patients (group 1) compared with patients who continued on PD (group 2) (p<0.05). No significant differences between outcome groups 1 and 2 in adjusted PDF levels of sC5b-9 were found in peritonitis caused by other kinds of microorganisms, with a limitation of small group sizes in these analyses (Fig 5A). In PDF of group 2 patients infected with *Staphylococcus aureus*, adjusted C3 was high differed from those infected with other microorganisms (Fig 5B). However, no significant differences were observed in adjusted C3 and C4 levels between groups 1 and 2 for individual microorganisms.

### Percent changes of sC5b-9 levels in PDF in relation to causative microorganisms

**Fig 6:**
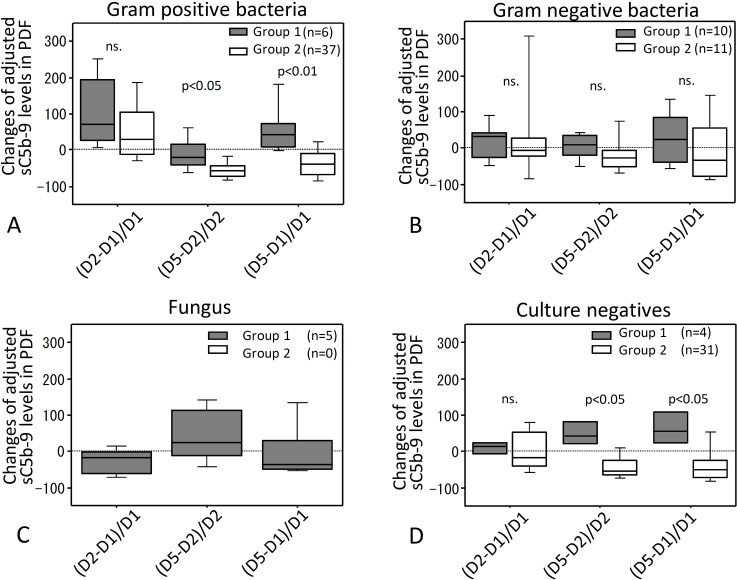
Percent changes of sC5b-9 levels in PDF in relation to causative microorganisms. PDF from cases were divided into four categories: gram positive bacteria (A), gram negative bacteria (B), fungal infection (C) and culture negative peritonitis (D). Percent changes of sC5b-9 levels were measured in each category. Gray filled hatched bar is group 1 and open hatched bar is group 2. ns: not significant. Statistical tests with Mann-Whitney U test and Bonferroni correction.

We focused on percent changes of adjusted PDF levels of sC5b-9 because the largest differences in a selected analysis between groups 1 and 2 were observed in adjusted sC5b-9 levels in PDF (Fig 3A). When causative microorganisms were divided into four categories: gram positive, gram negative, fungal and culture negative peritonitis, percent changes of levels of adjusted sC5b-9 in PDF between days 1, 2 and 5 of peritonitis varied depending on the categories of microorganisms (Fig 6). Percent changes in adjusted sC5b-9 levels in PDF from day 2 to day 5 and from day 1 to day 5 were significantly greater in group 1 than in group 2 when peritonitis was caused by gram positive bacteria or was culture negative (Fig 6A and 6D), but percent change of adjusted sC5b-9 levels in PDF were not different between the two groups in gram negative peritonitis (Fig 6B). Of note, percent changes of PDF levels of adjusted sC5b-9 in fungal peritonitis were apparently increased from day 2 to day 5 compared to from day 1 to day 2 and from day 1 to day 5. This phenomenon was not observed in other three categories in group 1 (Fig 6C). Regarding Fungus, we could not perform intergroup comparison because there were no fungal peritonitis cases in group 2.

### Usefulness of adjusted sC5b-9 levels and WBC counts in PDF to predict prognosis on day 5

When we compared levels of adjusted sC5b-9 levels in PDF with counts of WBC in PDF as a conventional peritonitis marker, a significant correlation was observed on days 1 and 5 (S1 Fig). However, when groups 1 and 2 were analysed individually, significant correlation was not found on any day in group 1 although significant correlation was still found on days 1 and 5 in group 2.

**Fig 7:**
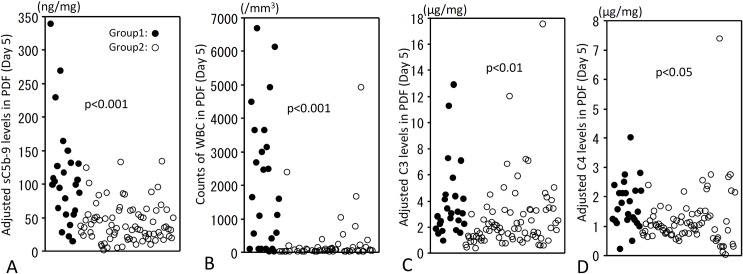
Levels of adjusted sC5b-9, C3 and C4 and WBC counts in PDF of peritonitis. Levels of adjusted sC5b-9 (A), counts of WBC (B), levels of adjusted C3 (C) and levels of C4 (D) in PDF are significantly different between groups 1 and 2 on day 5 after incidences of peritonitis. Statistical tests with Mann-Whitely U test and Bonferroni correction. Each filled circle is from individual case in group 1 and each open circle is from individual case in group2.

Focusing on day 5, levels of adjusted sC5b-9, adjusted C3 and adjusted C4 in PDF and counts of WBC in PDF were significantly higher in groups 1 than in group 2 (Fig 7). Therefore, a multivariate analysis was performed among these four measurements at this timepoint. Baseline imbalance was observed in PD history and albumin levels. These two variables were considered as adjusting factors and included in the logistic regression models (Table 3). In Model 1, WBC was a significant predictor of group 1 (OR per 100 units [95%CI], 1.125 [1.063, 1.194]; *P* < 0.001), in which c-index was 0.903.

**Table 3:** Logistic regression analysis for withdrawal caused by PD-related peritonitis on day 5.

Model	• Predictor • in PDF	• OR • 95% CI • *P*-value	c-index	• NRI • 95% CI • *P*-value	• IDI • 95% CI • *P*-value
Model 1	• WBC • (per 100)	• 1.125 • 1.060–1.194 • <0.001*	0.903		
Model 2	• WBC • (per 100)	• 1.080 • 1.016–1.147 • 0.013*	0.918	• 0.74 • 0.31–1.17 • <0.001*	• 0.05 • -0.01–0.12 • 0.126
	• sC5b-9 • (per 10)	• 1.262 • 1.049–1.518 • 0.014*			
Model 3	• WBC • (per 100)	• 1.126 • 1.054–1.203 • <0.001*	0.904	• 0.00 • -0.42–0.42 • 0.996	• 0.00 • -0.00–0.00 • 0.633
	C3	• 0.992 • 0.813–1.210 • 0.938			
Model 4	• WBC • (per 100)	• 1.113 • 1.048–1.182 • 0.001*	0.901	• 0.08 • -0.35–0.51 • 0.719	• 0.00 • -0.01–0.02 • 0.790
	C4	• 1.261 • 0.710–2.238 • 0.429			

Notes: PDF levels of sC5b-9, C3, and C4 were adjusted for protein levels in PDF. The logistic regression models were adjusted for PD history and serum albumin levels. PDF, peritoneal dialysis fluid; OR, odds ratio; CI, confidence interval; NRI, net reclassification improvement; IDI, integrated discrimination improvement; WBC, white blood cells.

*P < 0.05.

In Model 2, in which sC5b-9 was added to model 1, WBC and sC5b-9 were significant independent predictors of group 1. Odds ratios (95% CI; *P*-value) for WBC (per 100 units) and sC5b-9 (per 10 units) were 1.080 (1.016, 1.147; *P* = 0.013) and 1.262 (1.049, 1.518; *P* = 0.014), respectively. The c-index in model 2 was 0.918 which increased from that in Model 1. NRI showed significantly improved classificatory ability compared with Model 1. IDI, though not significant, showed a trend of improved classificatory ability compared with Model 1 (NRI [95% CI], 0.74 [0.31, 1.17], *P* < 0.001; IDI [95% CI], 0.05 [-0.01, 0.12], *P* = 0.126).

Meanwhile, in the models wherein C3 or C4 were added to Model 1, these factors were not statistically significant predictors of group 1, and the prediction capability was not improved either.

In the present study, optimal threshold of adjusted sC5b-9 value in PDF on day 5 to predict withdrawal, calculated as maximum value of the sum of sensitivity and false positive ratio, was 54.3 (area under the curve: 0.833, 95% CI: 0.730–0.936).

Temporal changes in levels of adjusted sC5b-9 and counts of WBC in individual PD cases in group 1 fitted one of the following 4 patterns: (A) levels of adjusted sC5b-9 increased, WBC decreased on day 5; (B) levels of adjusted sC5b-9 decreased or unchanged, WBC increased; (C) levels of adjusted sC5b-9 and WBC increased; (D) levels of adjusted sC5b-9 and WBC decreased (S2 Fig). Of note, the pattern (A) was observed in 36% of group 1 cases, suggesting that the combined use of WBC and levels of sC5b-9 in PDF might help to predict outcomes of peritonitis in PD patients.

## Discussion

In the present study, PD patients with peritonitis were divided into two groups that either failed to recover from peritonitis and were finally withdrawn from PD (group 1) or recovered from peritonitis (group 2). Protein-adjusted levels of sC5b-9, C3 and C4 in day 5 PDF of group 1 patients were significantly higher than those in group 2. Because the day 5 differences in levels of sC5b-9 were the largest differences observed between groups 1 and 2, we focused attention on sC5b-9 levels as a potential biomarker of outcome in PDF. When we assessed levels of sC5b-9 among four infection categories in PD-related peritonitis on day 5, PDF adjusted sC5b-9 levels in culture negative peritonitis and in peritonitis caused by gram positive bacteria were significantly higher in cases that progressed to withdrawal from PD (group 1) compared to recovery cases (group 2). We compared the changes of adjusted sC5b-9 levels in PDF between days 1 and 5 or between days 2 and 5 in these groups to ascertain whether changes in levels were more predictive; however, the results for most cases were similar to simply measuring adjusted sC5b-9 levels in PDF on day 5. We next separately assessed sC5b-9 levels in PDF from cases grouped by causative microorganism (including culture negatives) to assess whether this finding was organism-specific; adjusted sC5b-9 levels in PDF from group 1 cases were significantly increased compared with group 2 in culture negative peritonitis but in other groups failed to reach significance likely because of sample size limitations. Changes of sC5b-9 levels between days 1 and 5 or between days 2 and 5 were also significantly higher in group 1 compared to group 2 in gram positive and culture negative peritonitis. Sample size prevented statistical analysis for each species of the microorganisms.

The data show that levels of C proteins and activation products measured in PDF in peritonitis may be dependent on the causative microorganisms and sensitivity to the antibodies utilized; and further analyses of C markers might aid identification of the cause of the infection. Of note, in PDF with peritonitis caused by fungus or *Pseudomonas aeruginosa*, which had generally poor prognosis, all cases were withdrawn from PD (group 1), making it impossible to evaluate relevance of the marker to predict their prognosis; nevertheless, levels of sC5b-9 in PDF were similarly high in peritonitis caused by these organisms in group 1. Serum levels of C3, C4 and CH50 were not related to prognosis of peritonitis. We conclude that measurement of sC5b-9, and perhaps other C activation products in PDF might be helpful to predict prognosis of peritonitis in PD patients.

To diagnose PD-related peritonitis and follow the clinical course, WBC in PDF are generally measured [14,15]. In our study, levels of sC5b-9 in PDF were correlated with WBC counts in patients with peritonitis. However, levels of adjusted sC5b-9 and WBC in PDF were not correlated in patients that failed to recover from peritonitis (group 1). As shown in S2 Fig, various patterns of temporal change in adjusted sC5b-9 and WBC counts were observed in group 1. We thus speculated that measurement of sC5b-9 in PDF in addition to WBC counts, might be of more value than evaluation of only WBC in PDF. Our results of multivariate logistic regression support this hypothesis, suggesting that the model wherein sC5b-9 was added to WBC counts improved the predictive ability for withdrawal.

Peritonitis remains the biggest challenge to improving prognosis of PD [1,2,16]. The final decision to withdraw from PD is a difficult one for physicians because of remaining possibilities to control peritonitis by change of antibiotics. The recommendation of the 2010 ISPD guideline is to remove the PD-catheter if peritonitis remains uncontrolled on day 5 post-onset [3]. However, it is often difficult to know on day 5 whether peritonitis will persist; development of new surrogate markers may be helpful for physicians in making the final decision to remove the PD-catheter on day 5, a decision that precipitates surgical intervention and risk to the patient. The decision is particularly difficult when PDF bacterial cultures are negative but clinical peritonitis persists on day 5; both patients and physicians might hesitate to decide on PD-catheter removal and prefer a wait-and-see strategy that might be dangerous. In future, improved sensitivity of bacterial identification–for example, by analysis of mRNA fragments and immunological finger prints [17,18] might help; for the moment, our data suggest that measurement of levels of sC5b-9 in day 5 PDF may assist in making this difficult decision. High levels of sC5b-9 predict poor outcome and would support catheter removal while low levels predict recovery and would give cause for further delay and monitoring.

Our results suggest that levels of C activation products in PDF, especially sC5b-9, might be biomarkers to aid decision-making regarding treatment of PD patients with peritonitis, especially culture negative peritonitis. Combinations of sC5b-9, C3 and C4 and other complement marker levels in PDF might also give clues to the causative bacteria, further improving treatment and outcome. Measuring biomarkers in PDF has advantages because harvesting samples is easy and not harmful for patients. A limitation of the present study was that the causative bacterial panel was broad and cases of each microorganism were limited. In our results that culture negative peritonitis were mostly recovered with conventional antibiotics, culture negative peritonitis might be many times fastidious coagulase negative staphylococcus as previous reports described about culture negatives [19–21]. Of course, conventional antibiotics might be inadequate in some culture negative peritonitis cases of group 1. In future, analyses of the C system as markers might aid identification of the cause of the infection; it would be helpful to analyze larger groups of samples to support these results.

## Supporting Information

S1 Fig Correlation between levels of adjusted sC5b-9 and counts of WBC in PDF. (A), (B) and (C) show correlation between levels of adjusted sC5b-9 and counts of WBC in PDF in every cases on days 1, 2, and 5, respectively. (D), (E) and (F) show correlation between levels of adjusted sC5b-9 and counts of WBC in all cases in PDF in group 1 on days 1, 2, and 5, respectively. (G), (H) and (I) show correlation between levels of adjusted sC5b-9 and counts of WBC in PDF in group 2 on days 1, 2, and 5, respectively. Statistically, correlation between adjusted sC5b-9 and counts of WBC in PDF was significantly observed on days 1 (A) and 5 (C) when they were compared in all cases. However, when groups 1 and 2 were analysed individually, statistical correlation was not found on any day in group 1 (D, E and F) although significant correlation was still found on days 1 and 5 in group 2 (G and I).
(PDF)

S2 Fig Combination pattern between levels of adjusted C5b-9 and WBC counts in PDF in group 1. Each cases in group 1 were observed as four combination pattern between levels of adjusted sC5b-9 in PDF and counts of WBC in PDF on days 1, 2 and 5 after incidence of peritonitis as the following; levels of adjusted sC5b-9 in PDF increased on day 5 although counts of WBC decreased in PD (A), levels of adjusted sC5b-9 in PDF decreased or kept the same level on day 5 although counts of adjusted WBC increased (B), both levels of adjusted sC5b-9 and counts of WBC in PDF increased on day 5 (C), and both levels of adjusted sC5b-9 and counts of WBC in PDF decreased on day 5 (D).
(PDF)

S1 Table Background of the institutes which episodes with peritonitis were studied. (PDF)

S2 Table Original data of statistical analysis in the present study. (PDF)
