# Supplementary figures and images for: High Levels of Soluble C5b-9 Complex in Dialysis Fluid May Predict Poor Prognosis in Peritonitis in Peritoneal Dialysis Patients
Source: PLoS One. 2017 Jan 3;12(1):e0169111. doi: 10.1371/journal.pone.0169111 (PMC5207753; doi:10.1371/journal.pone.0169111)

# S1 Fig

Mizuno M, *et al.*

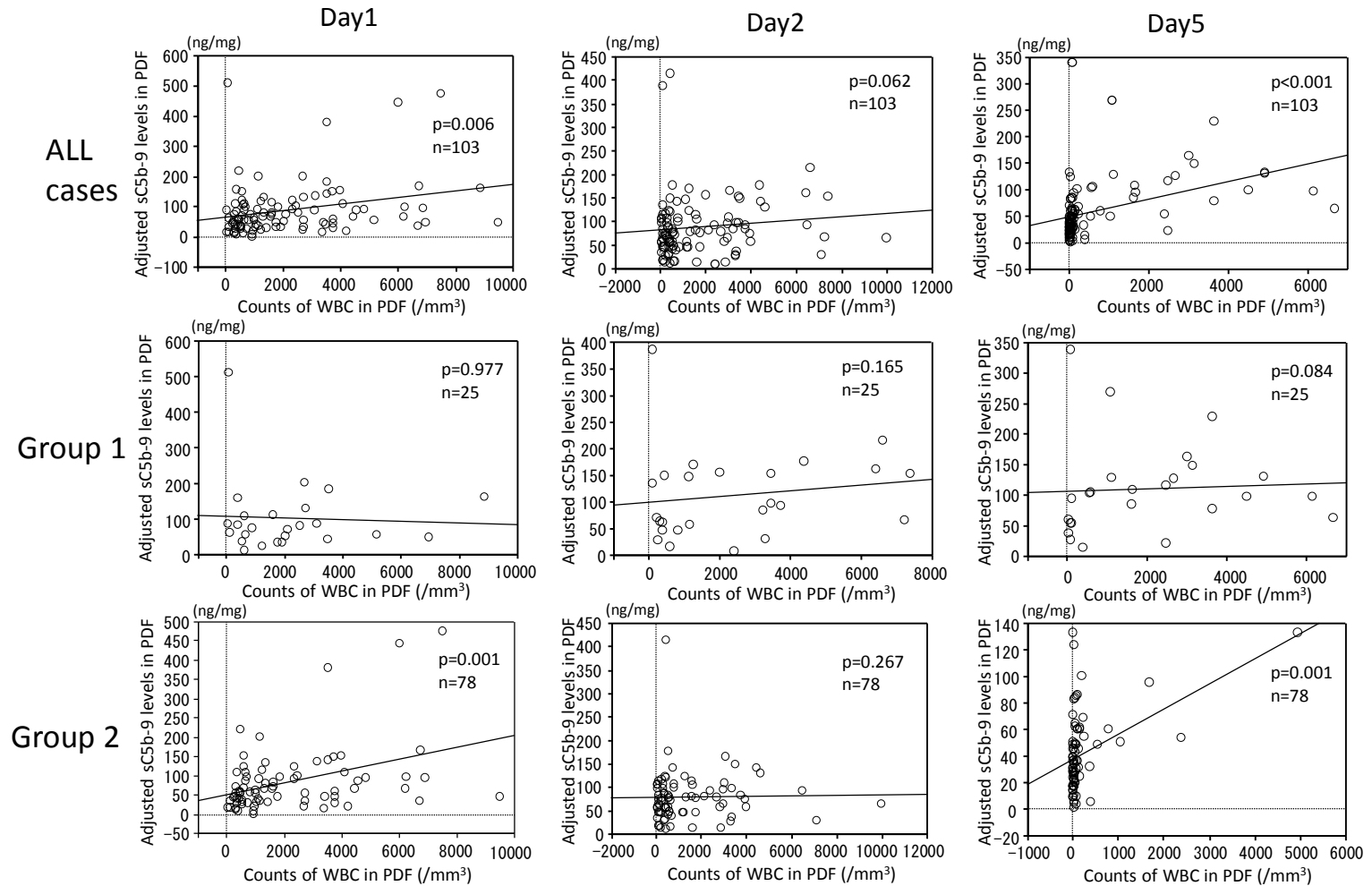

Supplement: S1 Fig — (A), (B) and (C) show correlation between levels of adjusted sC5b-9 and counts of WBC in PDF in every cases on days 1, 2, and 5, respectively. (D), (E) and (F) show correlation between levels of adjusted sC5b-9 and counts of WBC in all cases in PDF in group 1 on days 1, 2, and 5, respectively. (G), (H) and (I) show correlation between levels of adjusted sC5b-9 and counts of WBC in PDF in group 2 on days 1, 2, and 5, respectively. Statistically, correlation between adjusted sC5b-9 and counts of WBC in PDF was significantly observed on days 1 (A) and 5 (C) when they were compared in all cases. However, when groups 1 and 2 were analysed individually, statistical correlation was not found on any day in group 1 (D, E and F) although significant correlation was still found on days 1 and 5 in group 2 (G and I). (PDF) [file pone.0169111.s001.pdf]

# S2 Fig

Mizuno M, *et al.*

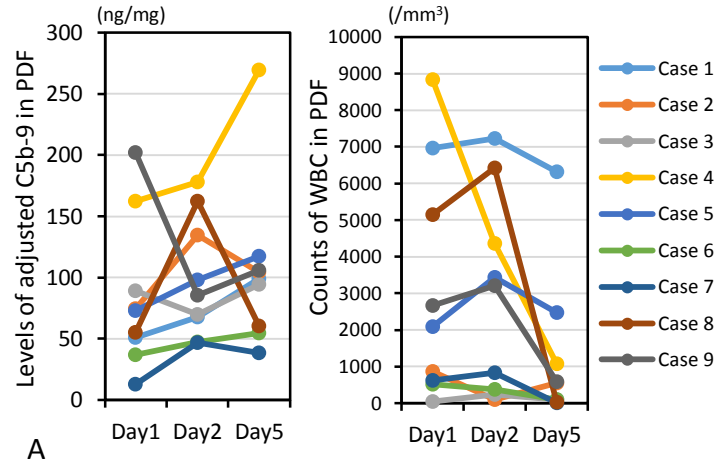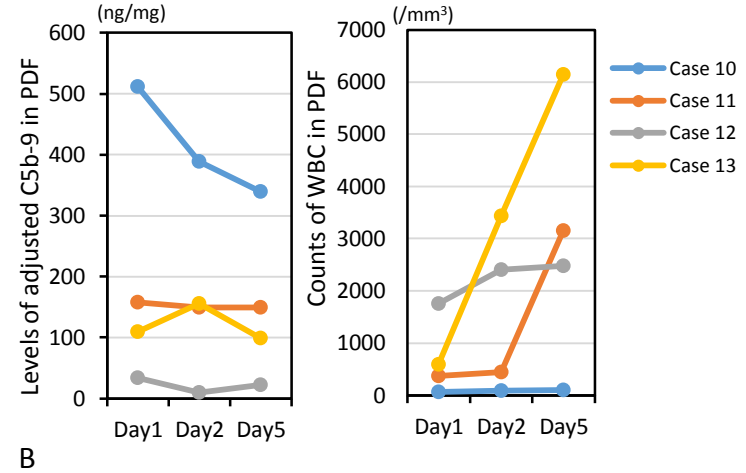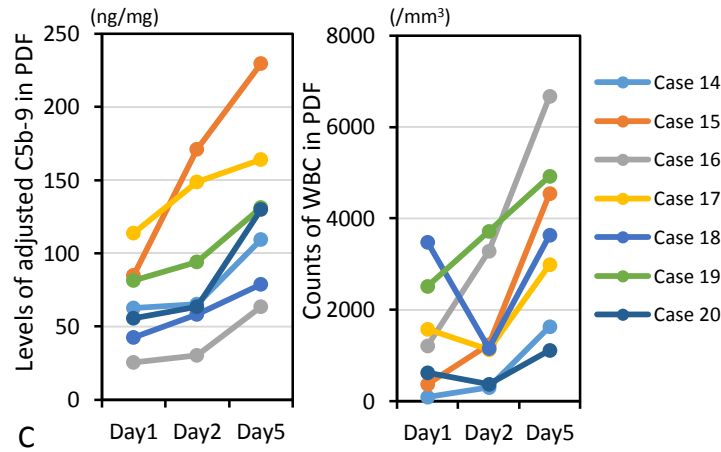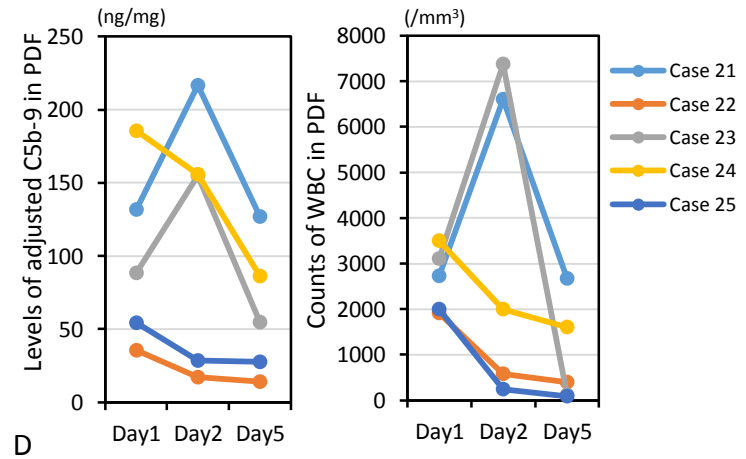

Supplement: S2 Fig — Each cases in group 1 were observed as four combination pattern between levels of adjusted sC5b-9 in PDF and counts of WBC in PDF on days 1, 2 and 5 after incidence of peritonitis as the following; levels of adjusted sC5b-9 in PDF increased on day 5 although counts of WBC decreased in PD (A), levels of adjusted sC5b-9 in PDF decreased or kept the same level on day 5 although counts of adjusted WBC increased (B), both levels of adjusted sC5b-9 and counts of WBC in PDF increased on day 5 (C), and both levels of adjusted sC5b-9 and counts of WBC in PDF decreased on day 5 (D). (PDF) [file pone.0169111.s002.pdf]
