# Supplementary material for: High Levels of Soluble C5b-9 Complex in Dialysis Fluid May Predict Poor Prognosis in Peritonitis in Peritoneal Dialysis Patients
Source: PLoS One. 2017 Jan 3;12(1):e0169111. doi: 10.1371/journal.pone.0169111 (PMC5207753; doi:10.1371/journal.pone.0169111)
Supplement: S1 Table — (PDF) [file pone.0169111.s003.pdf]

Supplementary Table 1 Background of the institutes which episodes with peritonitis were studied.

Terms/Institutes	Total number of PD patients (/observed term)	Number of episodes of peritonitis	Number of PD patients with peritonitis	Number of episodes of peritonitis to be used in the present study
2008.1~2008.12				
Nagoya University Hospital	47	4	4	1
Daiyukai Daiichi Hospital	99	33	26	17
2009.1~2009.12				
Nagoya University Hospital	50	5	5	0
Daiyukai Daiichi Hospital	73	26	15	20
2010.1~2010.12				
Nagoya University Hospital	53	7	6	0
Daiyukai Daiichi Hospital	70	13	10	13
2011.1~2011.12				
Nagoya University Hospital	59	6	6	2
Daiyukai Daiichi Hospital	66	27	20	23
2012.1~2012.12				
Nagoya University Hospital	54	4	4	2
Daiyukai Daiichi Hospital	63	26	19	17
Handa City Hospital	39	15	10	1
2013.1~12013.12				
Nagoya University Hospital	56	3	3	1
Daiyukai Daiichi Hospital	65	19	15	7
