# Supplementary material for: High Levels of Soluble C5b-9 Complex in Dialysis Fluid May Predict Poor Prognosis in Peritonitis in Peritoneal Dialysis Patients
Source: PLoS One. 2017 Jan 3;12(1):e0169111. doi: 10.1371/journal.pone.0169111 (PMC5207753; doi:10.1371/journal.pone.0169111)
Supplement: S2 Table — (PDF) [file pone.0169111.s004.pdf]

Supplementary Table 1

Dummy ID	Age	Group	Gender (male 1; female 0)	DM (1) or nonDM (0)	PD history (month)	Serum Alb level	Blood urea nitrogen level	Serum creatinine level	Serum C3 level	Serum C4 level	Serum CH50	Categories of causative microorganisms	TP in PDF (day 1)	TP in PDF (day 2)	TP in PDF (day 3)	WBC in PDF (day 1)	WBC in PDF (day 2)	WBC in PDF (day 5)	sC5b-9 in PDF (day 1)	sC5b-9 in PDF (day 2)	sC5b-9 in PDF (day 5)	sC5b-9 adjusted with TP (day 1)	sC5b-9 adjusted with TP (day 2)	sC5b-9 adjusted with TP (day 5)	C3 adjusted with TP (day 1)	C3 adjusted with TP (day 2)	C3 adjusted with TP (day 5)	C4 adjusted with TP (day 1)	C4 adjusted with TP (day 2)	C4 adjusted with TP (day 5)
1	64	2	1	0	3	3.5	37.0	12.53	84.0	20.0	37.6	culture negative	2757	2182	2099	2320	213	30	254.00	160.74	78.22	92.1	73.7	37.3	1.573	2.485	0.602	1.113	1.835	0.786
2	70	2	0	1	8	2.6	37.0	5.43	85.4	33.2	42.5	culture negative	3207	4386	3417	570	50	5	488.20	472.80	96.85	152.2	107.8	28.3	2.366	0.500	0.432	1.665	0.353	0.680
3	86	1	0	0	133	2.2	40.0	4.59	N/A	N/A	N/A	G−	2374	4456	3499	70	83	95	1213.50	1731.00	1188.00	511.2	388.5	339.5	3.197	4.528	1.882	1.968	2.110	1.254
4	75	2	1	0	28	4.0	41.0	8.34	N/A	N/A	N/A	G−	2489	2191	1923	400	1760	25	106.74	101.78	45.08	42.9	46.5	23.4	0.859	1.136	1.275	0.758	0.754	0.828
5	67	2	1	0	5	3.4	51.0	8.20	80.0	29.0	50.4	culture negative	2051	1845	1512	1130	35	10	413.50	196.50	52.50	201.6	106.5	34.7	3.926	2.635	1.190	1.170	1.100	1.084
6	77	2	1	1	47	3.8	40.0	12.41	N/A	N/A	N/A	G+	2150	2376	1881	33	2120	15	37.50	197.00	85.50	17.4	82.9	45.5	0.420	0.814	0.880	0.562	0.598	0.648
7	70	1	1	1	24	3.3	46.0	6.85	N/A	N/A	N/A	G−	2921	3008	3570	6960	7230	4490	149.00	203.50	354.00	51.0	67.7	99.2	1.354	1.548	2.801	1.644	1.939	2.409
8	71	2	0	1	33	3.3	39.0	6.27	94.0	32.0	42.7	G+	1909	1725	1449	7480	430	30	909.90	715.40	180.30	476.6	414.7	124.4	8.111	9.732	2.986	4.625	4.935	2.393
9	64	2	1	1	22	3.2	44.0	6.00	107.0	29.0	44.5	G+	3642	2986	2386	3410	2986	2386	177.00	328.50	128.50	48.6	110.0	53.9	1.672	1.347	1.257	1.501	1.290	1.189
10	73	2	1	0	52	2.3	78.0	8.80	N/A	N/A	N/A	G−	4509	6147	2664	3710	700	200	684.10	872.90	269.30	151.7	142.0	101.1	0.943	1.608	0.843	1.169	1.868	1.011
11	77	1	1	1	48	3.4	41.0	10.41	N/A	N/A	N/A	G+	1795	2338	3474	98	298	1635	112.00	152.60	379.20	62.4	65.3	109.2	0.632	0.952	1.483	0.730	0.714	1.193
12	65	2	1	0	8	3.7	36.0	7.92	84.0	20.0	37.6	G+	3016	1976	2048	1110	512	15	280.30	154.50	57.20	92.9	78.2	27.9	1.189	1.019	0.392	0.748	0.945	0.655
13	65	1	1	0	9	3.8	40.0	10.81	84.0	20.0	37.6	G+	2290	2242	2182	860	88	550	170.70	302.40	227.50	74.5	134.9	104.3	1.128	1.170	2.242	0.926	0.831	1.107
14	71	2	0	1	24	3.2	44.0	6.21	94.0	32.0	42.7	G+	2671	5053	2751	1000	537	3	151.20	427.00	111.20	56.6	84.5	40.4	2.525	3.273	1.241	2.377	1.872	1.329
15	74	2	1	0	32	3.0	40.0	5.32	N/A	N/A	N/A	G+	2080	3140	2615	933	2373	10	43.10	296.40	58.40	20.7	94.4	22.3	1.807	2.448	1.648	1.780	2.011	1.519
16	67	1	1	0	82	1.7	65.0	5.46	N/A	N/A	N/A	G−	2677	3375	2784	365	1240	3640	226.70	576.40	638.80	84.7	170.8	229.5	5.388	4.877	2.416	2.432	1.704	1.579
17	71	2	0	1	24	3.2	44.0	6.21	94.0	32.0	42.7	culture negative	3431	3028	2270	1000	537	3	223.10	177.00	103.70	65.0	58.5	45.7	1.537	1.595	1.104	1.524	1.142	1.055
18	76	2	1	0	34	3.8	39.0	8.05	N/A	N/A	N/A	culture negative	2264	2348	2124	520	413	70	77.80	134.00	105.20	34.4	57.1	49.5	0.991	1.836	1.482	0.791	1.002	1.027
19	75	2	1	1	79	3.5	60.0	9.27	61.0	18.0	32.0	G+	4993	2332	2632	2710	640	3	176.26	167.37	187.75	35.3	71.8	71.3	2.673	1.358	0.911	2.087	1.184	0.965
20	84	1	1	0	55	3.4	44.0	7.28	N/A	N/A	N/A	G+	4052	2168	2387	2720	6600	2670	533.54	469.42	303.01	131.7	216.5	126.9	3.056	2.370	1.693	2.499	2.255	2.134
21	75	2	1	0	55	4.0	76.0	9.36	98.0	29.8	42.4	G+	2785	1334	1786	2720	90	3	156.62	88.05	91.02	56.2	66.0	51.0	0.697	1.427	1.177	0.729	1.414	1.081
22	58	2	1	1	1	2.8	54.0	7.90	87.0	22.0	37.5	G+	4686	5332	2808	2333	415	28	579.65	643.97	233.87	123.7	120.8	83.3	3.775	5.095	2.569	2.668	2.847	1.688
23	62	2	1	0	55	3.5	62.0	14.35	N/A	N/A	N/A	G+	3178	3409	2970	1060	2830	120	130.07	196.10	137.22	40.9	57.5	46.2	0.875	1.086	0.919	1.097	1.338	0.998
24	79	1	1	0	34	3.2	114.0	15.01	N/A	N/A	N/A	culture negative	3752	5074	6011	1210	3280	6680	95.36	153.55	382.60	25.4	30.3	63.6	0.453	0.408	0.942	0.332	0.229	0.208
25	72	2	1	0	12	3.6	49.0	5.91	N/A	N/A	N/A	G−	4202	3426	1967	3950	3050	5	646.49	572.98	18.92	153.9	167.2	9.6	7.856	5.326	1.791	2.599	1.813	0.848
26	58	1	1	1	2	2.7	40.0	7.23	87.0	22.0	37.5	culture negative	4793	4552	5034	50	228	104	426.29	318.41	476.32	88.9	69.9	94.6	5.838	6.349	4.104	1.912	2.237	1.783
27	68	2	1	1	20	4.1	53.0	10.37	84.0	20.0	30.4	culture negative	5184	2978	2111	1030	150	10	175.23	58.45	14.85	33.8	19.6	7.0	5.478	5.494	3.549	1.337	1.147	0.931
28	57	1	1	0	105	2.8	74.0	14.08	50.0	14.0	22.7	culture negative	15227	3691	5633	8840	4360	1080	2468.86	656.95	1517.94	162.1	178.0	269.5	4.135	2.057	4.451	1.678	1.419	2.127
29	68	2	1	1	20	3.6	46.0	7.26	126.0	31.0	54.7	G+	1831	2049	2078	915	420	20	17.67	24.55	2.99	9.7	12.0	1.4	2.471	4.877	2.246	0.857	1.344	0.743
30	68	2	1	1	21	3.5	44.0	7.37	126.0	31.0	54.7	G+	2679	2025	1729	1425	35	18	88.29	72.66	18.30	33.0	35.9	10.6	2.661	2.602	1.658	1.013	0.830	0.764
31	69	1	1	1	69	2.7	62.0	9.83	67.0	24.0	19.9	G−	2298	3000	2913	2000	240	90	125.09	84.92	79.94	54.4	28.3	27.4	1.621	3.386	3.440	1.139	1.723	2.524
32	62	1	1	1	15	2.8	31.0	7.00	106.0	30.0	41.7	culture negative	2370	3637	6242	1580	1130	2990	269.94	541.32	1024.94	113.9	148.8	164.2	2.921	7.816	7.259	1.724	2.007	2.756
33	68	2	1	0	21	3.4	48.0	7.62	80.0	29.0	50.4	G+	5196	3390	2310	1610	65	8	434.34	331.88	112.64	83.6	97.9	48.8	5.054	6.582	4.500	1.834	0.909	0.800
34	75	2	0	0	28	3.6	37.0	8.14	97.0	29.0	50.4	G+	833	3066	1647	325	203	90	91.72	145.15	142.34	110.1	47.3	86.4	6.718	2.149	2.375	3.587	1.610	1.625
35	70	2	1	1	14	3.4	52.0	5.76	88.0	16.0	42.0	G+	3086	3192	2746	2420	620	70	316.69	52.67	12.36	102.6	16.5	4.5	2.986	2.784	3.140	1.027	0.902	0.851
36	89	2	1	0	46	3.3	62.0	6.33	105.5	32.1	49.0	culture negative	2659	3076	2510	1613	130	3	195.67	194.24	72.31	73.6	63.1	28.8	5.286	2.849	1.971	1.194	0.770	0.702
37	40	2	1	1	4	3.4	58.0	8.08	80.0	26.0	34.7	G+	5038	3488	2003	6230	308	0	499.44	345.78	36.30	99.1	99.1	18.1	9.398	8.707	2.179	2.169	1.329	0.725
38	59	2	1	1	0	3.6	53.0	7.83	114.0	34.0	58.6	culture negative	2326	2435	2013	643	20	3	204.94	139.69	78.37	88.1	57.4	38.9	3.265	3.047	2.104	1.623	1.570	1.232
39	69	2	1	1	26	2.5	65.0	14.30	N/A	N/A	N/A	G+	2902	2579	3086	633	310	55	324.38	232.39	152.17	111.8	90.1	49.3	3.754	3.477	1.709	1.023	1.959	1.032
40	89	1	1	0	49	3.2	54.0	6.33	105.5	32.1	49.0	G−	2969	3544	5507	2080	3440	2470	217.39	348.32	646.10	73.2	98.3	117.3	5.510	6.212	11.298	1.677	2.197	2.135
41	65	2	0	1	38	3.0	57.0	8.87	N/A	N/A	N/A	G+	2822	5108	3131	355	85	5	83.10	430.22	76.46	29.4	84.2	24.4	1.422	3.927	1.193	0.608	1.199	0.557
42	61	2	1	1	46	2.1	37.0	6.72	85.7	34.2	52.0	G+	2294	2616	2317	555	3480	18	164.15	389.69	63.50	71.6	149.0	27.4	2.644	10.391	1.934	1.484	1.875	1.182
43	61	2	0	1	39	2.9	50.0	8.55	N/A	N/A	N/A	G+	3844	4423	3277	1145	220	30	141.89	345.84	112.00	36.9	78.2	34.2	1.512	3.371	1.603	0.534	1.188	0.616
44	62	1	1	1	49	2.4	29.0	6.12	85.7	34.2	52.0	G−	2273	1905	2685	3480	1150	3640	97.50	111.33	211.32	42.9	58.4	78.7	2.070	2.994	2.673	1.484	1.243	1.207
45	61	1	0	1	41	2.5	54.0	8.59	N/A	N/A	N/A	G+	2706	3620	3640	515	370	90	100.37	171.46	199.69	37.1	47.4	54.9	2.931	3.090	3.013	1.392	1.549	1.405
46	68	2	1	1	33	3.5	58.0	9.61	126.0	31.0	54.7	G+	3293	2827	2415	6660	348	13	121.96	87.09	11.10	37.0	30.8	4.6	5.282	3.149	0.724	1.278	0.634	0.484

47	73	2	0	1	44	2.5	47.0	6.86	94.0	32.0	42.7	culture negative	7018	7319	4064	3360	1620	150	112.08	97.14	247.18	16.0	13.3	60.8	7.778	7.005	5.024	1.944	1.965	1.118
48	73	2	0	1	45	2.5	48.0	7.72	94.0	32.0	42.7	culture negative	1776	5626	3185	470	313	48	393.01	487.68	206.33	221.3	86.7	64.8	11.585	5.378	3.264	2.950	1.103	1.317
49	75	2	1	0	1	3.2	35.0	4.80	50.0	30.0	23.8	culture negative	2609	3123	1816	1315	400	3	211.33	100.46	241.54	81.0	32.2	133.0	3.560	5.663	0.770	1.608	1.814	0.747
50	50	2	0	1	45	3.5	63.0	10.44	108.0	34.0	46.3	culture negative	3252	4160	4337	400	7080	383	199.71	122.04	26.11	61.4	29.3	6.0	2.732	6.496	6.830	1.051	1.808	2.177
51	60	2	1	1	11	3.6	31.0	6.83	114.0	34.0	58.6	culture negative	4826	3374	2396	913	225	60	11.17	392.91	206.35	2.3	116.5	86.1	5.425	3.146	1.501	1.598	0.780	0.773
52	54	2	1	0	34	2.9	64.0	11.82	84.0	42.0	39.6	culture negative	3275	4003	3314	4070	550	38	361.53	711.90	277.91	110.4	177.9	83.9	10.327	13.509	12.072	3.205	3.770	1.587
53	66	2	1	0	22	2.1	39.0	7.40	128.0	34.0	58.6	culture negative	3973	2650	2464	4800	2690	95	377.42	209.50	89.94	95.0	79.1	36.5	8.566	5.299	3.094	3.400	2.266	1.094
54	60	2	1	1	12	3.3	39.0	7.34	114.0	34.0	58.6	culture negative	4042	5375	3094	690	775	40	402.99	541.90	63.33	99.7	100.8	20.5	3.869	4.682	1.265	1.424	1.601	0.739
55	67	2	1	0	10	2.7	47.0	11.12	133.0	30.0	47.6	culture negative	2973	3534	2024	255	68	5	144.88	68.87	35.00	48.7	19.5	17.3	5.237	2.286	1.071	2.000	1.079	1.120
56	60	2	1	1	4	4.0	68.0	5.55	106.0	30.0	49.6	culture negative	4953	2789	2330	3720	85	30	148.38	129.12	41.19	30.0	46.3	17.7	5.482	4.249	1.851	1.963	1.104	1.015
57	48	2	1	0	3	3.2	52.0	7.42	98.0	29.0	50.4	culture negative	4795	5272	4467	6880	3360	88	456.12	514.98	271.91	95.1	97.7	60.9	6.667	9.349	4.566	1.858	1.621	1.420
58	60	2	1	1	14	3.2	36.0	7.64	114.0	34.0	58.6	culture negative	4685	4864	3644	280	1760	115	343.12	380.91	114.22	73.2	78.3	31.3	3.883	3.812	2.280	1.401	1.638	0.923
59	54	2	1	0	37	2.8	59.0	12.29	84.0	42.0	39.6	G+	3267	3330	2918	N/A	1610	90	265.37	348.21	178.17	81.2	104.6	61.1	8.991	5.541	5.880	2.915	1.322	1.014
60	69	2	1	1	23	3.6	52.0	12.84	N/A	N/A	N/A	G+	2222	1523	3160	338	200	25	92.06	21.94	54.64	41.4	14.4	17.3	1.877	1.097	1.837	0.997	0.926	0.858
61	58	1	1	0	21	3.8	53.0	9.30	82.0	27.0	45.3	fungus sp	3742	4033	6585	375	440	3150	591.22	603.81	982.94	158.0	149.7	149.3	3.667	3.861	5.756	1.054	1.111	1.838
62	60	2	1	1	16	3.5	34.8	8.92	114.0	34.0	58.6	culture negative	2092	4113	2948	443	1540	65	119.31	340.22	108.41	57.0	82.7	36.8	1.663	4.732	1.002	1.106	1.502	0.724
63	67	2	0	0	9	3.2	51.0	6.64	136.0	43.0	50.8	G+	2189	2993	2805	315	3310	80	35.99	84.38	27.97	16.4	28.2	10.0	1.091	3.300	2.357	1.252	2.320	1.435
64	76	2	1	0	12	3.0	59.0	13.86	50.0	30.0	26.8	G−	1939	2913	2072	110	2870	3	34.51	40.15	66.27	17.8	13.8	32.0	1.664	1.523	0.825	1.516	1.009	1.039
65	70	2	1	1	26	3.6	41.0	13.46	N/A	N/A	N/A	G−	3634	4463	4171	2680	3940	235	82.88	339.38	228.65	22.8	76.0	54.8	2.430	3.471	2.980	2.156	2.299	1.513
66	66	2	1	0	28	2.7	38.0	10.35	128.0	34.0	58.6	G+	3133	4072	1708	210	38	0	139.12	235.82	53.05	44.4	57.9	31.1	2.552	3.170	1.542	1.069	1.365	1.114
67	56	2	0	0	144	2.2	72.0	7.62	N/A	N/A	N/A	culture negative	3236	3974	4063	783	520	1040	186.80	171.70	205.65	57.7	43.2	50.6	5.964	6.225	7.179	2.963	2.396	2.552
68	61	1	1	1	20	2.6	30.0	7.71	114.0	34.0	58.6	fungus sp	5290	6893	7015	1750	2410	2480	180.46	63.62	156.56	34.1	9.2	22.3	7.611	7.320	12.927	4.159	4.111	4.040
69	76	2	1	0	11	3.3	47.0	8.51	50.0	30.0	26.8	culture negative	2574	2950	2222	510	1150	8	73.63	136.03	32.13	28.6	46.1	14.5	2.101	2.163	1.144	1.658	1.803	1.258
70	73	2	0	1	60	3.4	49.0	7.60	94.0	32.0	42.7	G−	4190	6541	12133	1330	760	520	565.05	709.53	595.81	134.9	108.5	49.1	4.713	4.466	7.085	1.691	3.098	1.512
71	46	2	0	1	5	3.2	37.0	4.51	86.0	35.0	40.8	culture negative	3059	4679	4215	700	380	15	89.78	265.37	110.17	29.4	56.7	26.1	2.605	3.119	3.334	2.839	2.305	2.273
72	61	2	1	1	22	3.2	31.0	7.05	114.0	34.0	58.6	G+	3082	4431	3152	120	120	25	109.14	208.68	69.04	35.4	47.1	21.9	2.772	5.473	3.293	1.223	3.021	2.359
73	64	2	1	1	61	2.9	60.0	9.32	106.0	30.0	41.7	culture negative	3928	4468	5654	460	283	43	235.99	482.10	353.51	60.1	107.9	62.5	5.128	6.212	2.470	3.003	3.106	1.299
74	50	2	1	1	28	3.0	97.0	11.36	80.0	26.0	34.7	G+	3845	3549	2928	1750	95	3	187.25	410.55	52.10	48.7	115.7	17.8	5.739	5.823	1.368	0.303	2.995	1.343
75	46	2	0	1	6	3.4	29.0	3.68	86.0	35.0	40.8	culture negative	5107	6192	3298	9480	3750	20	245.67	528.06	84.59	48.1	85.3	25.7	4.817	3.754	1.583	3.431	3.022	2.772
76	55	1	1	0	46	2.9	67.0	13.40	84.0	42.0	39.6	G+	1820	2308	2848	623	825	15	23.94	108.28	109.57	13.2	46.9	38.5	1.385	1.820	4.330	0.395	0.386	0.508
77	75	2	1	0	47	3.1	76.0	6.58	84.0	42.0	39.6	G+	2714	2340	1697	3730	665	5	167.96	95.95	16.16	61.9	41.0	9.5	3.160	2.692	1.221	0.233	0.286	0.000
78	56	2	0	0	149	2.9	66.0	7.27	N/A	N/A	N/A	G−	3546	4453	2806	6000	2940	N/A	1580.50	287.32	246.45	445.7	64.5	87.8	4.545	2.783	3.221	1.049	0.916	0.573
79	55	2	1	0	10	3.8	52.0	8.03	86.0	27.0	53.6	G−	2479	3615	2237	310	70	53	140.27	271.68	96.84	56.6	75.2	43.3	1.359	2.282	1.582	1.388	1.065	0.872
80	71	2	1	0	6	3.7	48.0	7.76	115.0	22.0	45.7	G+	1900	1483	2272	4210	3330	150	39.06	56.05	57.56	20.6	37.8	25.3	1.132	0.782	0.612	0.553	0.566	0.502
81	78	2	0	0	64	3.0	28.0	9.86	97.0	29.0	50.4	culture negative	2561	2747	2239	1540	N/A	8	173.88	63.98	34.53	67.9	23.3	15.4	1.797	0.641	0.576	0.844	0.513	0.540
82	71	2	1	0	2	3.0	32.0	4.42	68.0	26.0	32.6	G−	4432	3229	2926	3490	9940	780	1691.22	208.62	176.90	381.6	64.6	60.5	3.890	1.533	1.094	1.931	0.815	0.615
83	81	2	0	0	20	2.8	36.0	8.78	98.0	41.0	39.3	G+	6399	3987	1321	3490	1535	1665	917.07	454.08	126.26	143.3	113.9	95.6	2.379	1.066	17.562	1.436	1.186	7.403
84	71	1	1	0	4	2.5	27.0	5.72	68.0	26.0	32.6	G−	2437	3502	3374	2510	3720	4920	198.15	329.55	442.82	81.3	94.1	131.3	1.410	1.325	1.748	0.931	1.042	1.285
85	72	2	1	0	10	3.4	29.0	8.46	115.0	22.0	45.7	G−	2159	2825	2360	393	4000	55	23.87	162.81	66.15	11.1	57.6	28.0	0.556	0.683	0.551	0.398	0.330	0.308
86	73	2	1	1	50	2.5	78.0	9.05	92.0	21.0	43.5	G+	3035	3938	3251	510	440	13	121.15	227.81	76.37	39.9	57.9	23.5	3.093	5.228	4.602	0.344	0.199	0.305
87	63	2	1	0	78	2.2	60.0	9.59	86.0	30.0	35.3	culture negative	6576	13434	4402	3140	2970	213	908.02	1284.05	304.50	138.1	95.6	69.2	8.406	10.488	3.285	0.231	0.070	0.107
88	75	2	0	0	99	3.7	74.0	7.54	N/A	N/A	N/A	G−	3863	3471	3306	6700	4420	4930	651.53	498.15	441.44	168.7	143.5	133.5	6.509	6.024	4.253	0.407	0.335	0.310
89	67	2	1	0	45	3.3	49.0	9.45	128.0	34.0	58.6	G+	2052	2932	2059	310	1230	13	29.64	139.20	35.44	14.4	47.5	17.2	1.184	2.022	0.918	0.522	1.770	0.024
90	65	2	1	0	8	3.8	58.0	7.58	98.0	27.0	50.5	G+	5295	2671	2845	4550	1290	15	466.42	211.58	100.68	88.1	79.2	35.4	6.241	2.708	3.495	2.914	1.458	1.153
91	71	2	1	1	61	3.6	47.0	8.13	98.0	28.0	42.0	G+	4217	5491	3023	1210	373	3	494.40	680.14	110.11	117.2	123.9	36.4	3.685	3.710	1.923	1.270	0.997	0.865
92	73	1	1	1	51	2.0	89.0	9.82	92.0	21.0	43.5	G−	3244	3460	3139	1910	580	400	114.93	59.89	43.73	35.4	17.3	13.9	2.541	3.420	3.140	1.320	1.217	1.212
93	75	1	0	0	100	2.4	57.0	7.61	N/A	N/A	N/A	G−	3237	3197	2696	3100	7380	108	286.58	495.35	147.68	88.5	154.9	54.8	7.753	6.171	2.486	2.574	2.098	2.195

94	82	1	1	1	87	3.7	44.0	11.43	91.0	34.0	40.7	G+	4451	4333	3785	5150	6420	18	246.83	702.81	228.05	55.5	162.2	60.3	3.044	2.928	1.573	2.145	1.487	1.097
95	56	1	1	0	20	3.6	52.0	8.40	86.0	27.0	53.6	G−	2736	4619	4544	590	3440	6140	298.45	716.71	447.59	109.1	155.2	98.5	4.269	6.428	7.075	2.636	2.391	1.485
96	57	1	1	0	27	3.4	78.8	7.69	86.0	27.0	53.6	fungus sp	4016	3541	3351	2670	3210	580	810.89	302.47	354.42	201.9	85.4	105.8	3.650	2.189	3.017	1.532	0.806	0.997
97	69	1	1	1	85	3.3	52.1	9.58	107.0	29.0	44.5	fungus sp	2596	2620	4174	625	365	1110	144.20	166.33	543.05	55.6	63.5	130.1	1.922	2.266	4.157	1.561	1.635	2.806
98	32	1	1	0	34	4.0	62.7	19.36	102.0	33.0	70.9	fungus sp	2605	4233	4078	3500	2000	1600	483.28	659.72	352.49	185.5	155.9	86.4	2.034	2.885	2.237	1.864	2.516	2.198
99	49	2	1	0	54	3.4	43.0	10.07	83.7	30.0	40.8	G+	3236	5683	3661	3740	4630	150	149.72	747.14	226.08	46.3	131.5	61.8	8.665	14.861	4.024	3.127	3.685	2.666
100	68	2	1	1	40	3.0	38.0	8.63	78.4	30.4	43.0	G+	3570	7267	3168	1810	6480	370	354.71	676.03	104.02	99.4	93.0	32.8	11.409	10.295	3.437	2.910	3.762	2.755
101	46	2	1	0	12	3.2	55.0	9.99	77.0	26.6	31.1	culture negative	7007	7443	5837	580	600	10	867.80	372.10	128.90	123.8	50.0	22.1	12.525	5.127	5.018	4.003	2.335	2.220
102	48	2	0	0	72	3.8	38.0	12.35	103.0	29.2	N/A	G+	2525	4880	2743	1290	1260	80	167.96	605.18	84.60	66.5	124.0	30.8	2.437	4.100	2.282	0.622	0.461	0.399
103	61	2	1	1	2	3.4	43.0	10.07	65.4	23.0	53.5	culture negative	5652	4944	3099	6170	570	50	386.24	389.16	60.92	68.3	78.7	19.7	3.777	3.074	1.718	0.681	0.492	0.315
104	65	2	1	0	3	2.8	61.0	10.12	75.5	21.8	42.0	culture negative	5169	6403	4399	4410	600	50	342.64	309.04	219.70	66.3	48.3	49.9	4.077	3.360	2.469	3.503	3.276	2.164
